# Can Poetry Be Used as an Educational Tool to Improve Understanding of Psychosis?

**DOI:** 10.1192/bjo.2025.10296

**Published:** 2025-06-20

**Authors:** Esther Okhiria, Pirashanthie Vivekananda-Schmidt

**Affiliations:** 1 Sheffield Health and Social Care, Sheffield, United Kingdom; 2 The University of Sheffield, Sheffield, United Kingdom

## Abstract

**Aims:** Mental health disorders are a leading cause of disability and mortality globally, with the UK burden rising post COVID-19. Increasing understanding of mental health is key to better care. We advocate for using poetry to communicate mental health experiences. Integrating art with science, as shown by Carvalho, da Fonseca and de Melo Tavares, 2021, enhances emotional growth and self-awareness. Muszkat et al., 2010, found poetry reading fosters empathy in medical students.

Psychosis, often linked to schizophrenia, can be difficult to relate to. Hence, the first author aimed to create a poetry collection to deepen understanding of the phenomenon.

**Methods:** To construct poems, a library of 15 patient experience videos on psychosis was curated. They were analysed and key themes were identified for clinician learning. Poetic condensation was used to create a collection of 7 poems covering themes identified.

To assess the external validity of the poems, 40 participants spanning medical students, academic lecturers, and junior doctors were recruited. Participants read the collection and provided anonymous feedback via a questionnaire. Participants rated statements on a 5-point Likert scale and provided free-form comments.

**Results:** **Sample poem:**

Herring.

*It* started with moving ground.

Melting objects and furniture

An itch I couldn’t reach

Spiders I couldn’t rid.

Waiting to be salvaged

One day I was fish in a car

drowning when no one was swimming.

That’s when I knew

‘Herring’ showcases hallucinations and delusions experienced during psychotic episodes and incorporates themes of isolation and fear.

**External validity:** One participant had personal experience of psychosis and reported that the collection accurately reflected their experience.

Among those with clinical experience, 80% agreed the poems depicted psychosis accurately. 75% of participants reported increased understanding, and 90% would recommend the collection. Some participants expressed interest in exploring the themes further in a workshop setting.

**Conclusion:** This study developed an evidence-based poetry collection on psychosis, using a method that allowed for patient voices to be centred while minimising damage to their mental wellbeing. Presenting knowledge in this way is also useful for engaging the public in recognising the signs of psychosis and understanding their loved one’s experiences. Incorporating medical humanities, allows for novel and creative ways of information assimilation. Future work more directly assessing the impact on empathy in medical trainees would be beneficial.

